# Effects of an Electric Field on the Conformational Transition of the Protein: A Molecular Dynamics Simulation Study

**DOI:** 10.3390/polym11020282

**Published:** 2019-02-07

**Authors:** Zhouting Jiang, Le You, Wenhui Dou, Tingting Sun, Peng Xu

**Affiliations:** 1 Department of Applied Physics, China Jiliang University, No. 258 Xueyuan Street, Xiasha Higher Education Zone, Hangzhou 310018, China; leyou1110@163.com (L.Y.); douwh1221@163.com (W.D.); Xupeng@cjlu.edu.cn (P.X.); 2 Department of Physics, Zhejiang University of Science and Technology, No. 318 Liuhe Road, Hangzhou 310023, China; Tingtingsun@zust.edu.cn

**Keywords:** molecular dynamics simulation, electric field, conformational transition

## Abstract

The effect of the electric field on the conformational properties of the protein 1BBL was investigated by molecular dynamics simulations. Our simulation results clearly capture the structural transitions of the protein sample from helix to turn or random coil conformation induced by the increasing strength of the electric field. During our analysis, we found that the conformational stability is weakened, and the protein sample is stretched as an unfolded structure when it was exposed in a sufficiently high electric field. The characteristic time when the jump occurs in the time evolution curves of root mean square deviation (RMSD) and radius of gyration *R_g_* decreases with increasing electric strength, which demonstrates the rapidly conformational transition that occurs. The number of intra-protein hydrogen bonds, which is the key factor for stabilizing the protein structure, is related to the overall size of the protein. The value of the dipole moment and characteristic time are both influenced by the strength, but are independent of the direction of the external field. The protein sample becomes rotated with the electric field direction. These conclusions provide a theoretical realization of understanding the protein conformational transition in an electric field and the guidance for anticipative applications.

## 1. Introduction

The groundwork in the field of protein folding demonstrated that the tertiary structure of the protein is in a unique state with the global minimum of the free-energy landscape of a protein [1]. However, due to any intrinsic or extrinsic factors or agents, the misfolding and aggregation of non-native protein structures are observed [2,3]. Despite the huge amount of studies on the protein folding-unfolding process, the inherent mechanisms and their relationships to experimental observables are still a challenge for the scientific community [4,5]. Beyond the traditional studies on protein structure and stability based on temperature [6,7], pH variations [8], and denaturants [9], exotic conditions such as pressure [10] and electric fields [11,12] provide new ways to understand the protein unfolding process. In recent years, several novel food-processing techniques have been developed, such as high-pressure processing, pulsed electric field processing, and electro-hydrodynamics [13], but their application into industries is still in its nascent stage. Therefore, the research on the effect of the electric field applied to proteins is not only important as a scientific issue, but also very meaningful under industrial applications. 

The protein response to an external field is involved in protein folding, protein adsorption, the protein recognition process, etc. [14,15,16,17]. Living systems naturally produce weak electric fields. Electrostatic forces, as long-range interactions, play a crucial role in defining the structure and properties of proteins [18,19,20]. Experimental studies have been carried out to understand the effect of an external environment on the protein properties, and even to explore the possibilities to control protein process via surface-enhanced resonance Raman spectroscopy (SERRS) [21], quartz crystal vibrational analysis (QCV) [22], the measurement of electrical double layer capacitance [23,24], etc. Although these methods could partly provide useful information about protein behaviors under an applied potential, difficulties are usually encountered when simultaneously applying electric fields and measuring protein behavior due to the limitation of measurement techniques [17,25]. For example, a nominal electrical field between 10^3^–10^8^ V/m was applied in the previous experimental works on protein responses to electric fields [14,16,21,26]. However, the exact nature and extent of field exposure on biological systems is difficult to measure. Some experimental results are not consistent because of the intrinsic difficulty of ensuring homogeneous field application. As a result, the research studies on proteins exposed in the electric fields are in an ongoing debate, and some phenomena are still not well understood. 

With the rapid development of computer technology, computational biophysics has started to focus on researching the structure and interactions of biomolecules at the atomistic level. Computational simulations provide an atomistic level description of protein behavior that cannot easily be observed from experiments [27]. It becomes one of the powerful methods to discover the inherent mechanisms of molecular biology. Recently, meaningful research has been carried out on the thermodynamics and dynamics of the protein by both internal and external factors, such as the properties of the protein, surface, solvent, confinement, thermal fields, and electric fields by molecular dynamics (MD) or Monte Carlo (MC) simulations [28,29,30,31,32,33,34]. Budi et al. have performed MD simulations on the insulin chain under the influence of both static and oscillating electric fields. They found that the oscillating fields are more disruptive to the structure than static fields with similar strength [28]. Long-time MD simulations were carried out to study the structural integrity of insulin under the external static fields. They reported that the secondary structure of insulin is disrupted by the external field with the strength of 0.25 V/nm [29]. The MD simulations were also performed to investigate the effects of external pulsed and static electric fields on the conformational properties of myoglobin [30]. The authors observed that myoglobin undergoes a fast unfolding process under relatively high electric fields by MD simulations. They detected that the root mean square deviation of myoglobin under the application of a 0.1 V/nm electric field was not distinguishable compared to no-field conditions. However, another study on a short alanine-based peptide was shown that applying the electric field with the intensity of 0.1 V/nm was capable of perturbing peptide conformation by involving the localized dipolar alignment [31]. It does leave the open question of the electric effects on susceptible forms of the protein. Although these research studies have investigated the effects of the external field on proteins and peptides, most of these bio-macromolecules are exposed under the relatively low electric strength. We know the globular proteins are classified by the Structural Classification of Proteins (SCOP) database including all-α, all-β, α+β, and α/β types [35]. The previous research studies are mainly focused on the protein with both an α-helix and β-sheet on its secondary structural level. In order to give a contribution to comprehending the conformational transition, especially the evolutions of α-helical structures of the protein, we have selected dihydrolipoamide dehydrogenase classified into all-α proteins, which comprises two *α*-helix fragments without *β*-sheets in its initial conformation, as a case study.

## 2. Simulation Details and Methods

In the current study, we have carried out MD simulations to explore the effect of external electric fields on a single protein molecule. The E3-binding domain of the dihydrolipoamide dehydrogenase from the 2-oxoglutarate dehydrogenase (2-OGDH) complex of *Escherichia coli* was adopted as a protein sample in the present work. 2-OGDH is one of prominent members of the primary energy-producing pathways of glycolysis and the tricarboxylic acid cycle [36]. The starting configuration of the objective protein was obtained from Protein Database Bank (PDB) with entry code 1BBL classified as all-α proteins according to the SCOP database. 

The protein molecule 1BBL consists of 37 amino acids residues. Hydrogen atoms were added on the protein, and histidine (His) residues were treated as the neutral state (HSE). The protein has a net positive charge. Then, one chlorine ion was added to the system to neutralize it. The protein configuration was enclosed in the center of a periodic cubic simulation box. The size of the simulation box is about 5.5 × 5.5 ×5.5 nm^3^. A total of 4413 TIP3 waters were filled in the simulation box. All of the simulations were performed using molecular dynamic algorithms implemented in an NAMD2.6 software package (Beckman Institute, University of Illinois at Urbana-Champaign, Urbana, IL, USA) with an all-atom CHARMM27 force field [37,38]. The system was first energy minimized with a converging criterion of maximum force value of 10 kJ/nm/mol using the steepest descent method for 60,000 steps. Then, 100 ps equilibrations were carried out at the constant temperature and pressure ensemble (NPT). The MD simulations were run in 30 ns at a constant temperature *T* = 310 K and constant pressure of *P* = 1 atm. The time step and grid spacing during the simulation were set to two fs and 0.1 nm, respectively. The van der Waals interactions were calculated by a switch potential with a switching function starting at a distance of 1.0 nm and reaching zero at 1.2 nm. The long-range electrostatic interactions were calculated by the particle mesh Ewald (PME) method. The protein configuration was subjected to external electric fields with different strengths. The strength of electric fields was adopted from −0.7 V/nm to 0.8 V/nm. Most of the electric fields were applied along the *z*-direction. In order to show the effect of the electric field orientation, some simulation results were obtained from 1BBL under the electric fields along the *x* or *y* directions with similar strength. The values of electric strength that were selected in our simulation are commonly used in other literatures [17,39,40]. One MD simulation was run without an external electric field as a reference. 

The root mean square deviation (RMSD) is an important tool that is used to characterize the conformational changes of proteins. It is defined as:

(1) $$RMSD=\sqrt{\frac{1}{N}\sum_{i=1}^{N}{\left|r_{final}(i)-r_{initial}(i)\right|}^{2}}$$

where *N* is the number of protein atoms, and *r_final_*(*i*) and *r_initial_*(*i*) are the coordinates of an atom *i* in its final structure and initial structure, respectively. The radius of gyration *R_g_* is a basic measurement of the overall size of a chain molecule. The change in the structure of a protein during MD simulations can be quantified by radius of gyration *R_g_*, which is defined as:

(2) $$R_{g}=\sqrt{\frac{1}{N}\sum_{i=1}^{N}{\left|r(i)-r_{center}\right|}^{2}}$$

where *N* is the number of protein atoms, and *r*(*i*) and *r_center_* are the coordinates of an atom *i* and the center of mass, respectively. In general, proteins possess an electric dipole moment by virtue of their structure constructed by some charged amino acids, such as lysine, arginine, aspartic acid, etc. Once an external electric field is applied on the protein, it induces a realignment charge with respect to the direction of the electric field. The dipole moment is defined as:

(3) $$\vec{d}=\sum_{i=1}^{N}q_{i}(i){\vec{r}}_{i}$$

where *N* is the total number of protein atoms, *q_i_* is the charge of the atom *i*, ${\vec{r}}_{i}$ is the directional vector of each atom, and the relation $d^{2}=d_{x}^{2}+d_{y}^{2}+d_{z}^{2}$ holds.

In the present work, the conformational stability of the protein during the simulation procedure was examined by calculating the root mean square deviation (RMSD). The radius of gyration was also analyzed to represent the conformational changes of the protein. The components and total value of the dipole moment are evaluated to study the orientation of the protein. The detailed transition of the secondary structure of the protein 1BBL was analyzed by the STRIDE algorithm. Also, the contribution of hydrogen bonds, including intra-protein and water–protein types, was also evaluated to show the correlation with the size of the protein. The results obtained from this study attempt to provide an insight into the structural transition of 1BBL with the effect of external electric fields at the molecular level, and ultimately, a practical possibility of applying external electric fields in biotechnological applications.

## 3. Results and Discussions

### 3.1. Root Mean Square Deviation (RMSD)

**Figure 1:**
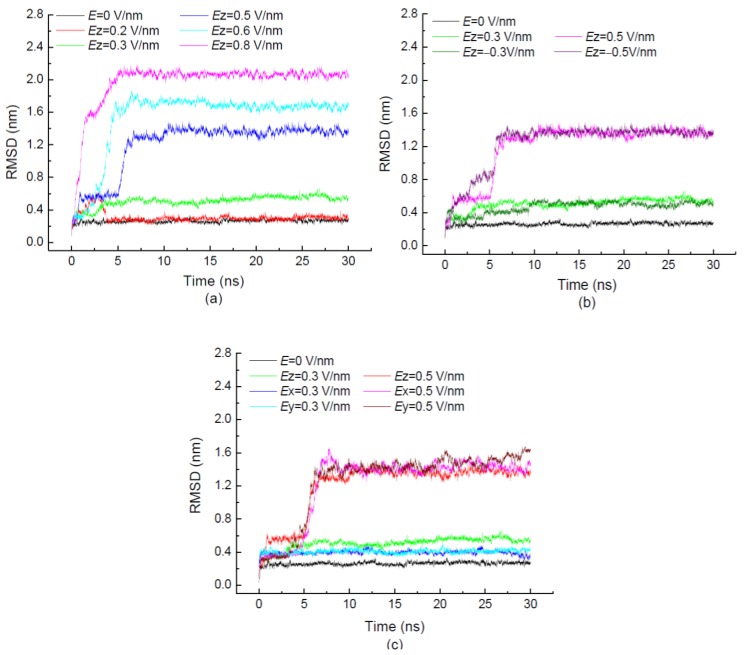
(Color online) Time evolution of root mean square deviation (RMSD) of the protein 1BBL exposed in the external electric fields with different strengths (**a**), along opposite *z*-directions (**b**), and along three different directions (**c**), respectively.

The time evolutions of RMSDs of 1BBL exposed in the applied electric fields with different strengths, and the different electric directions are shown in Figure 1. The figures indicate that the protein sample 1BBL reaches the equilibrium state within 10 ns simulations. In Figure 1a, the simulation results were obtained from 1BBL exposed in the electric field along the *z*-direction with different strengths. The value of RMSD increases to 0.6 nm when 1BBL is exposed in the low electric field with *Ez* = 0.2 V/nm for 3 ns, and then decreases to 0.3 nm, which has the approximate deviation of the protein sample in the absence of an electric field. It shows the effect of an external electric field on the stability of the protein. Meanwhile, the electric strength is not high enough to change the structure of 1BBL. With the increasing intensity of electric fields, the average value of RMSD over a period from 10 ns to 30 ns increases from 0.27 nm to 2.06 nm. Figure 1a also presents the visible jumps in the RMSD values when 1BBL was exposed in the electric fields higher than 0.5 V/nm, which indicates the appearance of conformational changes during the simulation process. The characteristic time at which the jump occurs in the curve of RMSD is located at 5.5 ns, 3.8 ns, and 1.3 ns in the condition of electric strength *Ez* = 0.5 V/nm, 0.6 V/nm, and 0.8 V/nm, respectively. It decreases with the increasing strength of the external fields. The change of characteristic time concludes that a sufficiently strong field leads to quickly destabilizing the conformation of the 1BBL protein. 

In order to investigate the effect of electric directionality on the protein sample, the simulations were performed with the same intensity of electric fields applied along opposite *z*-directions in Figure 1b, and along three different directions in Figure 1c. Figure 1b,c present the values of RMSD with the same intensity of the electric field applied in different directions. The average value of RMSD is increasing with the strength of the electric field. However, it is independent of the direction in which the external field was applied. It means that the protein 1BBL was rotated by the electric field. The obvious RMSD jumps during the simulation process are shown in the simulation case with the electric strength *E* ≥ 0.5 V/nm. The same characteristic time indicates that the structural change of the protein is related to the strength rather than the direction of the electric field. 

### 3.2. Radius of Gyration (R_g_)

**Figure 2:**
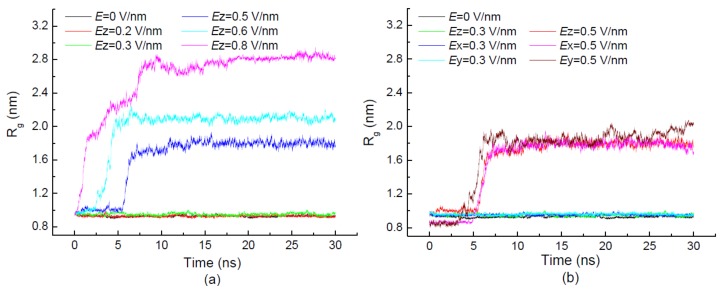

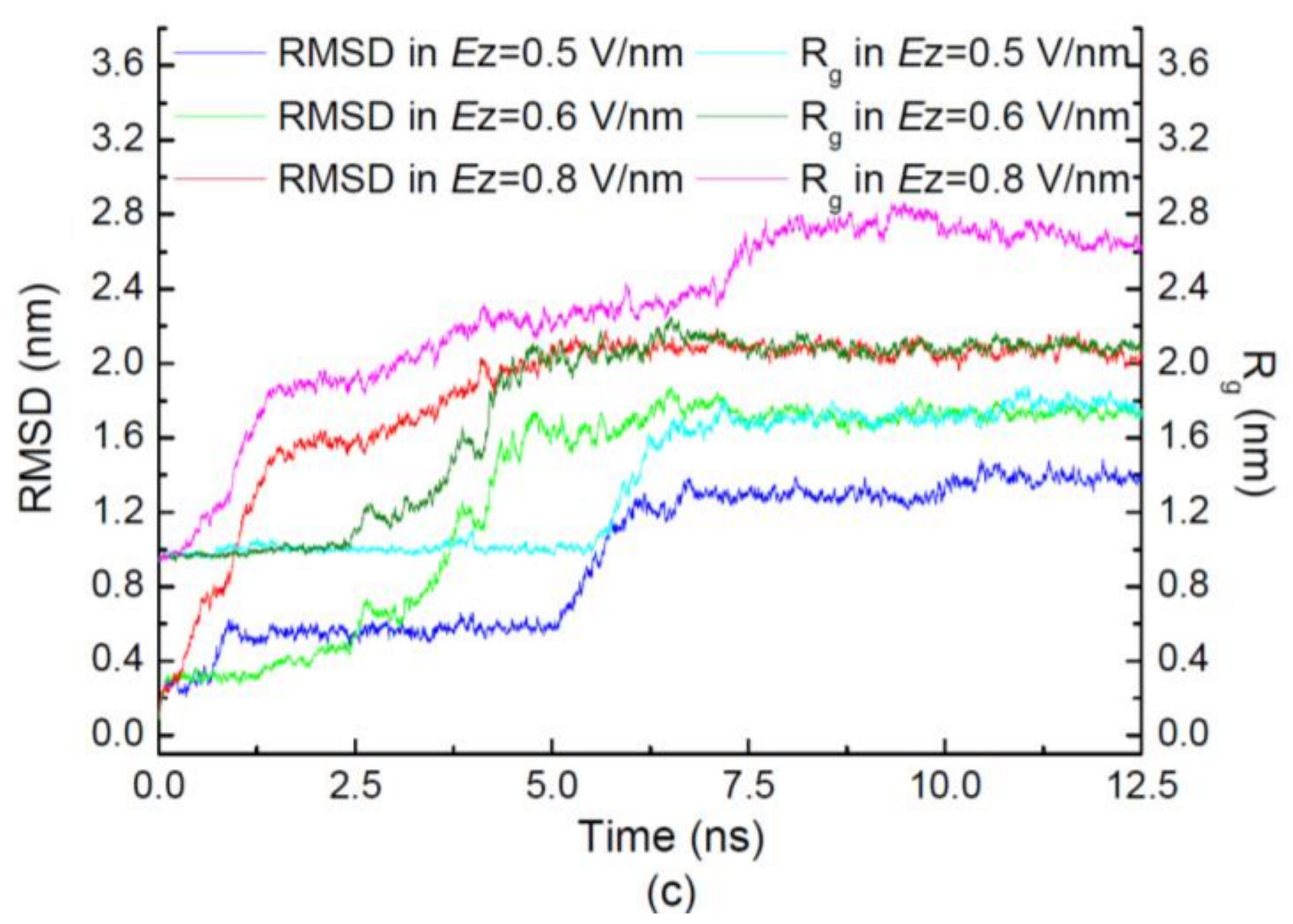
(Color online) Time evolution of radius of gyration *R_g_* of the protein 1BBL exposed in the external electric fields with different electric strengths (**a**), along three different directions (**b**), and the numerical comparison of *R_g_* and RMSD (**c**), respectively.

The time evolution of the radius of gyration of 1BBL exposed in the applied electric fields with different strengths and different directions are shown in Figure 2. Figure 2a,b show that the average value of *R_g_* slightly increases with the strength of electric field *E* ≤ 0.3 V/nm. With the intensity of electric field continuing to increase, the values of *R_g_* become more and more remarkable when 1BBL is exposed in the electric fields where *E* ≥ 0.5 V/nm. The reason partly lies in the stretching of the protein along the direction in which the electric field is applied. The average value of a radius of gyration *R_g_* over the simulation period from 10 ns to 30 ns increases from 0.93 nm to 2.78 nm with the increasing strength of the external field along the *z*-direction in Figure 2a. The visible jumps in the *R_g_* values also occur within 10 ns during the simulation process. It indicates the conformational transition of the 1BBL sample when it was exposed in the sufficiently high electric field. Figure 2a also shows that the characteristic time at which the jump occurs in the curve of *R_g_* decreased with increasing electric strength. It presents the same tendency as the time evolution of RMSD values in Figure 1a. The short characteristic time of *R_g_* indicates that the rapidly conformational transition of the protein 1BBL happened. 

To further investigate the effect of the electric directionality on the protein sample, the external fields along the *x*, *y* or *z* directions were adopted as the different simulation conditions. Figure 2b clearly presents the approximate average values of *R_g_* when 1BBL was under the electric fields with the same intensity but different directions. Even the similar characteristic time of *R_g_* is dependent on the strength rather than the direction of the electric field. It also indicates that the stretch ratio of 1BBL is strongly related to the intensity of the electric field. Meanwhile, the overall size of the protein has not changed greatly because of its rotation along the same direction as the electric field that was applied.

To explicitly demonstrate how the electric field induces the structural transformation of the protein, the characteristic times of RMSD and *R_g_* obtained from the initial 12.5-ns simulation processes are compared in Figure 2c. The characteristic time decreases with the increasing strength of the external electric field, indicating that the protein 1BBL changes its configuration more rapidly in the higher electric field condition. It also can be seen in Figure 2c that the sharp increase of RMSD is earlier than the curve of *R_g_* when the protein sample exposed in the electric field with the strength *Ez* = 0.5 V/nm. With the increasing strength of the electric field, the difference between the characteristic time of RMSD and *R_g_* is negligible. The protein has quickly destabilized the conformation and adjusted to new one under the sufficiently strong electric field.

### 3.3. Secondary Structure Analysis

The protein 1BBL consists of 37 amino acids that can be classified into all-α proteins, which comprises two alpha-helix fragments (No. 15-23 residues and No. 41-47 residues) in its initial conformation. The effect of the external electric field on the secondary structure of the protein 1BBL was estimated by the STRIDE algorithm implemented in the VMD software package (Beckman Institute, University of Illinois at Urbana-Champaign, Urbana, IL, USA) [41]. It helps to simplify the analysis of the tertiary conformation of a protein by assigning different types of secondary structure to each residues based on the knowledge-based algorithm, which takes into account the hydrogen bond energy and statistically derives the information on the torsional angles of the protein. 

**Figure 3:**
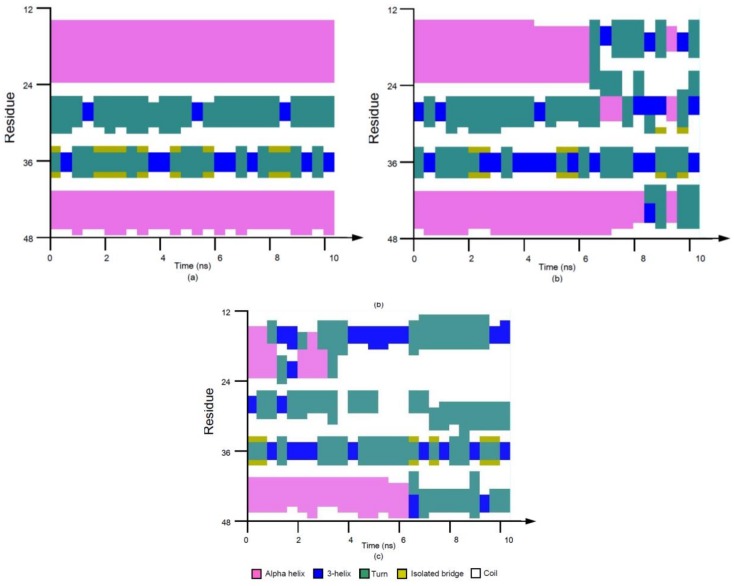
(Color online) Stride evolution of secondary structures of the protein 1BBL exposed in the external electric fields along the *z*-direction with the strength (**a**) *Ez* = 0 V/nm, (**b**) *Ez* = 0.5 V/nm, and (**c**) *Ez* = 0.6 V/nm, respectively.

The stride evolutions of secondary structures of 1BBL with different electric field strengths in the initial 10-ns MD simulation processes are shown in Figure 3. The time evolution of the secondary structure was obtained from the protein sample 1BBL, which was subjected to the electric field applied along the *z*-direction with the strength *Ez* = 0.5 and 0.6 V/nm (Figure 3b,c) and *E* = 0 as the reference state (Figure 3a). Focusing on the residues from No. 15 to 23 (labeled as fragment I) and No. 41 to 47 (labeled as fragment II) in Figure 3a, most residues are assigned a purple color, which indicates that the secondary structure of these two fragments remain alpha helices during the simulation process performed without an external electric field. With the effect of the external electric field, secondary structural transitions of 1BBL occur in both fragment I and II (see Figure 3b,c). As seen in the graphs, the fragments I and II, which originally formed as helical regions (purple), deconstruct as turns or random coils (cyan or white color). The conformational change in fragment I is earlier and more drastic than that in fragment II. Comparing the time evolution of the secondary structure in Figure 3b,c, the simulation results clearly present that the time when the structural transition starts decreases with the increasing strength of the applied electric field. The tendency is as same as the time evolution curve of the RMSD and *R_g_* shown in Figure 1 and Figure 2. The secondary structural analysis in Figure 3 also presents that the number of amino acids involved in the formation of turns and random coils increases with the increasing strength of the external electric field. It means that the stretch of the protein by an external field leads to destabilizing the conformation. This behavior can be attributed to the despiralization of the helix structure to the turns and random coils when the protein was subjected to an electric field.

**Figure 4:**
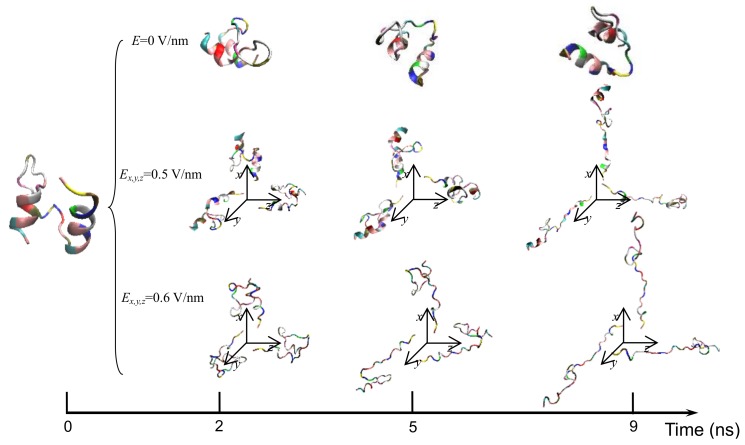
(Colored) Typical conformation of the protein 1BBL exposed in the electric fields with the strength *Ex,y,z* = 0 V/nm, 0.5 V/nm, and 0.6 V/nm during the simulation process, respectively.

The typical conformations of the protein sample 1BBL exposed in the electric fields along the *x*, *y*, or *z* direction with various strengths *Ex,y,z* = 0 V/nm, 0.5 V/nm, and 0.6 V/nm are shown in Figure 4. MD simulations were carried out from the same initial configuration, including two alpha-helix secondary fragments. In the reference state without an external field, the 1BBL conformation basically remains as the original structure with occasional orientation during the simulation process. When 1BBL is exposed in the electric field with relatively strong strength, the protein was stretched as the realignment of some charged residues with respect to the direction of the electric field. With the increasing strength of the electric field, the despiralization of the alpha-helix structure to the turns and random coils becomes more rapid and drastic. After the reconstruction of the unfolded structure, the protein kept its new state with stable RMSD and *R_g_* values. When the protein 1BBL was exposed in the electric fields with the same strength but different directions, the conformation is kept when the protein is orientated by changing the direction of the electric field. It confirmed the simulation results shown in Figure 1 and Figure 2. That is, almost the same values of RMSD and *R_g_* are obtained when the protein 1BBL is exposed in the electric fields with the same strength but different directions due to the orientation of the protein sample.

### 3.4. Dipole Moment

**Figure 5:**
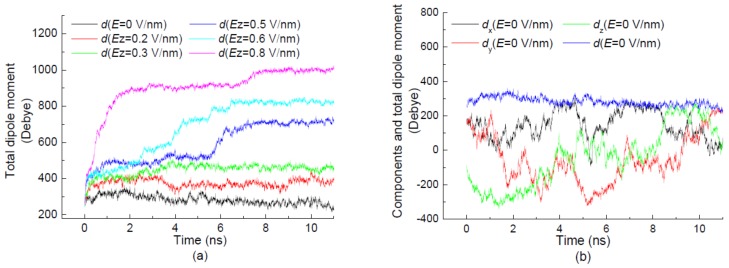

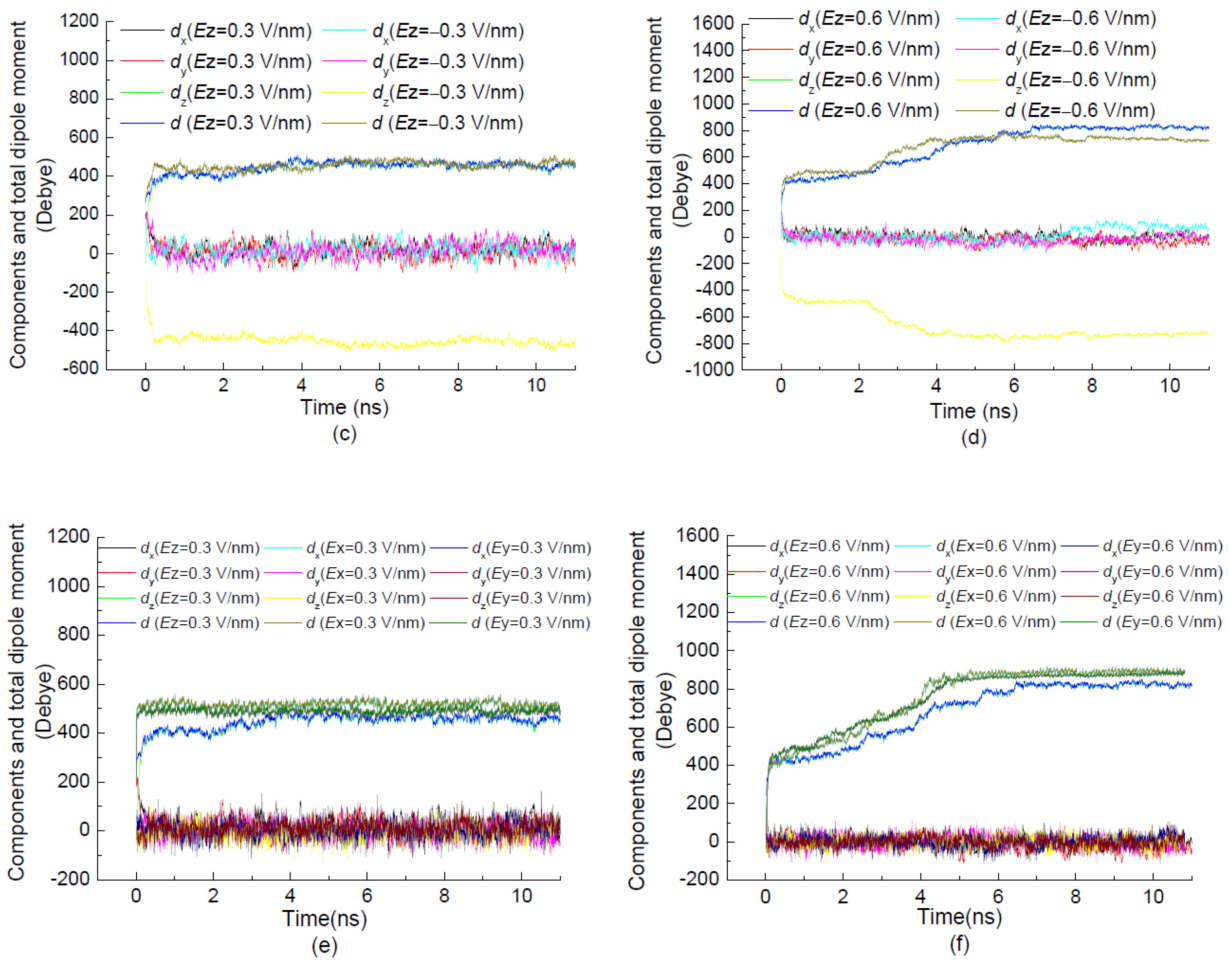
(Color online) Time evolution of total dipole moment of the protein 1BBL exposed in the electric fields along the *z*-direction with different strengths (**a**), and three components of the dipole moment in the condition of electric strength (**b**) *E* = 0 V/nm, (**c**) *Ez* = ±0.3 V/nm, (**d**) *Ez* = ±0.6 V/nm, (**e**) *Ex,y,z* = 0.3 V/nm, and (**f**) *Ex,y,z* = 0.6 V/nm, respectively.

With the effect of electric fields applied along different directions, the three components and total dipole moment of 1BBL as a function of the simulation time are shown in Figure 5. Figure 5a presents the time evolutions of the total dipole moment during the initial 10-ns simulation when the protein sample was exposed in the electric fields and without the electric field as the reference state. As seen in Figure 5a, a significant rise is observed in the curve of the total dipole moment with the increase of the electric strength. The general effect of applied electric fields on the dipole moment is to increase its magnitude, whose extent becomes more pronounced in the higher electric fields. The curves also present the good coherence with the tendency of RMSD and *R_g_* changing over the simulation time. The visible jumps in the curve of the total dipole moment are observed when 1BBL was exposed in the electric field with the strength *Ez* ≥ 0.5 V/nm. The time at which the jump occurs during the simulation process decreases with the increasing strength of the applied electric field. It means that the higher strength of the electric field induces the earlier achievement to the plateau of the total dipole moment.

The effects of the strength and direction of the external electric fields on three components of the dipole moment are compared in Figure 5b–f. When the simulation was carried out in the absence of the electric field condition, the three components of the dipole moment show obvious fluctuations between positive and negative values. Meanwhile, the value of the total dipole moment is about 275 D, with relatively stable values in Figure 5b. In the reference state without an external field, the amplitude of such fluctuations is much larger than that of the protein exposed in the electric field (see Figure 5c–f). In Figure 5c,d, when the electric field was applied along the positive *z*-axis, the *x* and *y* components of the dipole moment alter between positive and negative values randomly, regardless of the electric strength. The *z* component of the dipole moment remains a positive value. Apparently, when the protein exposed in the electric field along the negative *z*-direction, the *z* component of the dipole moment presents negative values. It is also shown in Figure 5e,f that the total dipole moments have approximate values with the component ones along the same direction of the electric field. It means that the component of the dipole moment with the same direction of the electric field is dominated in the value of the total dipole moment. The other two components perpendicular to the electric field direction oscillate between positive and negative values, with the average value close to zero. The average values of the total dipole moment increase with the intensity of the electric field. Meanwhile, the average values of the total dipole moment are independent of the electric direction, which is mainly due to the orientation of the protein by the charged atoms arranged along the same direction as the applied electric field. 

### 3.5. Hydrogen Bonds (HBs)

**Figure 6:**
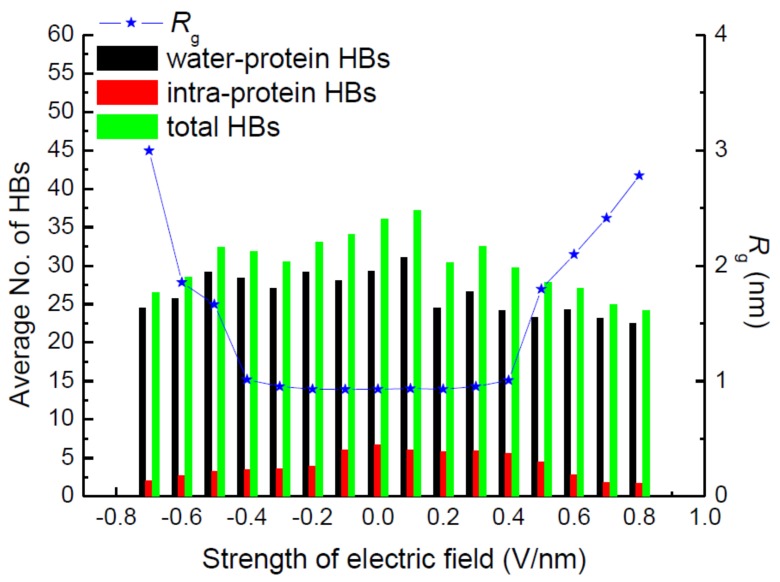
(Color online) Average number of hydrogen bonds (HBs) with respect to the radius of gyration *R_g_* of the protein 1BBL exposed in the external fields along the *z*-direction with different electric strengths and opposite directions.

Hydrogen bonds (HBs) play a significant role in stabilizing the secondary structure of proteins. Then, the correlation of hydrogen bonds with structural parameters such as *R_g_* was analyzed under the various simulation conditions. Figure 6 presents the electric strength dependence of the average number of total HBs, including intra-protein and water–protein types. The average numbers of HBs are the mean of the data over the last 10-ns MD simulations. As shown in Figure 6, the average number of water–protein HBs is several times higher than that of intra-protein HBs. Then, the change of the total number of HBs is dominated by the numerical variation of water-protein HBs. Regarding the view of the electric strength dependency of intra-protein HBs, the clear trend that the average number decreases with the increasing amplitude of the electric field is observed in Figure 6. It demonstrates that the number of intra-protein HBs presents a closer relation with the structural properties of the protein. The structural stability mostly depends on the interactions between amino acids in the formation of secondary structures via HBs, especially intra-protein HBs. Comparing the curve of radius of gyration *R_g_* and the number of average intra-protein HBs as the function of different electric strengths, the opposite trend is observed, i.e., the increasing value of *R_g_* is corresponding to the decreasing number of intra-protein HBs simultaneously when the protein is exposed in the higher electric field, which manifests the deconstruction of intra-protein HBs when the protein is stretched along the same direction as the electric field that is applied. The lower value of *R_g_* could be attributed to the construction of HBs involving intra-protein atoms. It verifies that HBs, especially intra-protein types, play the vital effect on promoting the stability of the protein. 

## 4. Conclusions

In this work, the effects of external electric fields with different strengths and directions on the protein 1BBL were investigated by MD simulations. The conformational characteristic and structural stability of the protein were discussed according to our simulation results. It concluded that the application of an external electric field could increase the value of root mean square deviation and the radius of gyration. The increases of overall size of the protein are attributed to the stretch along the electric direction. The protein sample is unfolded by the sufficiently strong electric field. The average values of RMSD and *R_g_* are dependent on the strength rather than the direction of the electric field, indicating the rotation of the protein sample along the same direction as the electric field that was applied.

When the protein is exposed in the electric field with strength *E* ≥ 0.5 V/nm, visible jumps of RMSD and *R_g_* are observed during the simulation process. It indicates the secondary structural transitions from helix to turn or random coil as long as the strength of the external electric field is sufficiently strong. The characteristic time when the jump occurs in the time evolution curves of RMSD and *R_g_* decreases with as the electric strength increases. The short characteristic time indicates the rapidly conformational transition. The characteristic time is also strongly related to the electric strength, but independent of the electric direction when the protein sample was exposed in the electric field. 

The dipole moment analysis showed that the external electric field could affect the 1BBL conformation by changing the axial component of the dipole moment. The magnitude of the dipole moment along the same direction of the electric field rises more rapidly and more intensively with the increase of electric strength. The higher fields have a more remarkable effect on the dipole moment. The value of the total dipole moment is mainly contributed by the axial component as the electric direction. It is due to the orientation of the protein sample by the charged atoms arranged along the same direction of the electric field. The conclusions achieved from MD simulation will be helpful to understand the effect of external electric fields on the conformational change of a protein, and provide guidance for anticipative applications.
